# Association between the Chinese Dietary Inflammatory Index and risk of gastric cancer: a case-control study in Southeastern China

**DOI:** 10.3389/fnut.2025.1653575

**Published:** 2025-08-19

**Authors:** Xinyu Chen, Yuhang Chen, Zhijie Luo, Lu Cheng, Qingying Wang, Fengqin Zou, Yulan Lin

**Affiliations:** Fujian Provincial Key Laboratory of Environment Factors and Cancer, Department of Epidemiology and Health Statistics, School of Public Health, Fujian Medical University, Fuzhou, China

**Keywords:** gastric cancer, dietary inflammatory index, dietary nutrients, Chinese adults, case-control study

## Abstract

**Objective:**

This study aimed to investigate the association between the Chinese Dietary Inflammatory Index (CHINA-DII) and the risk of gastric cancer (GC) among adults in Fujian Province of China.

**Methods:**

A 1:1 matched case-control study was conducted between July 2023 and November 2024. A total of 336 newly diagnosed GC cases were recruited from the Union Hospital, and 336 sex-matched healthy controls were enrolled from communities in Fujian Province. Dietary data were collected using food frequency questionnaire (FFQ), and conditional logistic regression models were used to assess the association between CHINA-DII scores and GC risk.

**Results:**

A total of 672 participants were included, comprising 336 GC cases and 336 controls. The proportions of males and females were 56.5 and 43.5%, respectively. The mean age of the case group was 56.76 ± 10.34 years, significantly higher than that of the control group (53.86 ± 11.13 years, *P* < 0.001). The average CHINA-DII score was −2.11 ± 0.62. Multivariable logistic regression analysis showed that higher intakes of vitamin C (OR = 0.69, 95% CI: 0.50–0.95) and vitamin D (OR = 0.67, 95% CI: 0.48–0.92) were significantly associated with lower GC risk. Higher CHINA-DII scores were positively associated with increased GC risk (OR = 1.45, 95% CI: 1.05–1.99), and each 1-standard-deviation increase in the CHINA-DII score was associated with a 1.26-fold increase in GC risk (OR = 1.26, 95% CI: 1.07–1.48). Subgroup analyses revealed significant positive associations between CHINA-DII and GC risk among individuals aged ≤ 55 years (OR = 2.44, 95% CI: 1.51–3.96), the married population (OR = 1.41, 95% CI: 1.01–1.96), non-smokers (OR = 1.70, 95% CI: 1.14–2.54), and those with high levels of perceived daily stress (OR = 2.82, 95% CI: 1.67–4.75).

**Conclusion:**

Lower intake of dietary vitamin C and vitamin D, as well as a higher overall dietary inflammatory potential, were significantly associated with an increased risk of GC. Younger, non-smoking, and those under greater psychological stress may be more sensitive to dietary inflammation.

## 1 Introduction

Gastric cancer (GC) is one of the most common malignant tumors worldwide and poses a serious threat to human health. GLOBOCAN 2022 reported 968,000 new GC cases and 660,000 deaths globally (5th highest cancer incidence/mortality) (1), with projections suggesting worsening burden by 2050 (2). China accounts for 37.02% of global GC cases (358,000 annually) and 39.44% of deaths (1). China's GC hotspots include Fujian Province, where incidence reaches 28.31/100,000 with distinct clustering in coastal cities like Putian (3–5). GC accounts for 9.3% of local cancers and ranks third in mortality (20.88/100,000) (4).

GC typically develops through chronic inflammation triggered by Helicobacter pylori, smoking, and dietary factors like high-salt and preserved foods (6). These promote tumorigenesis by creating a pro-inflammatory microenvironment that drives malignant transformation (7, 8).

Diet is a modifiable risk factor for GC, and unhealthy dietary patterns such as high intake of processed meats and foods high in salt can increase risk (9–12). In contrast, diets rich in antioxidant-containing foods such as fruits and vegetables, particularly those high in vitamin C, folate, and carotenoids, may exert protective effects by modulating metabolic pathways (13). However, most previous studies have focused on individual dietary components or nutrients, limiting the ability to evaluate the synergistic effects of multiple dietary factors on overall dietary inflammatory potential.

The Dietary Inflammatory Index (DII) was first proposed by Cavicchia et al. (14) and further refined by Shivappa et al. (15) to quantify the pro- or anti-inflammatory potential of an individual's diet. This scoring system integrates the inflammatory effects of 45 dietary components and was developed based on dietary intake data from 11 countries. It has been widely applied in research on chronic diseases such as diabetesand cardiovascular disease (16).

In recent years, several studies have investigated the association between DII and GC risk. European Prospective Investigation into Cancer and Nutrition (EPIC) cohort study found that each one–standard deviation increase in DII was associated with a 1.25-fold higher risk of GC. Moreover, individuals in the highest DII quantile had a 1.66 times greater risk of developing GC compared to those in the lowest quantile (17). Another population-based prospective cohort study reported that a one-quantile decrease in DII was associated with a reduced risk of GC (HR = 0.73, 95% CI: 0.53–0.99) (18). However, evidence from Chinese populations remains limited and inconsistent. For example, a case-control study conducted in Anhui Province observed a positive, though not statistically significant, association between DII and precancerous gastric lesions (OR = 1.34, 95% CI: 0.78–2.32) (19). In contrast, a study from Xinjiang found a significantly increased risk of esophageal cancer among individuals with high DII scores (OR = 2.55, 95% CI: 1.61–4.06) (20).

This inconsistency may be partly attributed to the original DII being primarily developed based on Western dietary data, which may not adequately capture the structure and inflammatory characteristics of Chinese diets. To address this limitation, our team has developed a localized version of the index—the Chinese Dietary Inflammatory Index (CHINA-DII)—based on dietary intake data from Chinese populations (21). The CHINA-DII has undergone reliability and validity assessments and is better suited to reflect the inflammatory potential of typical Chinese diets (21).

In summary, the main objective of this study is to assess the association between CHINA-DII and GC risk in a Chinese population and to explore its potential value in the primary prevention of gastric cancer.

## 2 Methods

### 2.1 Study design and study participants

A 1:1 sex-matched case-control study was conducted in Fujian Province, China, involving participants who met predefined inclusion and exclusion criteria. All participants were aged 18–75 years and were residents of Fujian, defined as having lived in the study area for at least 6 months within the 12 months preceding the survey.

The case group consisted of newly diagnosed patients with GC confirmed by histopathological or cytological examination between February and December 2024 at Fujian Medical University Union Hospital. The inclusion criteria for cases were: (1) aged 18–75 years; (2) resident of Fujian Province (residing in the study area for ≥6 months within the past year); (3) able to communicate effectively; and (4) provided written informed consent and voluntarily participated in the study. Exclusion criteria for cases included: (1) history of any cancer; (2) pregnancy or lactation; and (3) extreme daily energy intake (females >3,600 kcal or < 500 kcal; males >4,200 kcal or < 600 kcal).

The control group consisted of healthy residents recruited during the same period from nine prefecture-level cities in Fujian Province, matched by sex. Inclusion criteria for controls were: (1) aged 18–75 years; (2) resident of Fujian Province (residing in the study area for ≥6 months within the past year); (3) able to communicate effectively; and (4) provided written informed consent and voluntarily participated in the study. Exclusion criteria for controls were: (1) any history or current diagnosis of malignant tumors; (2) presence of major diseases (e.g., stroke or psychiatric disorders); and (3) extreme daily energy intake (females >3,600 kcal or < 500 kcal; males >4,200 kcal or < 600 kcal).

The sample size is calculated using the following formula: where M is the total number of pairs to be investigated; m is the number of pairs with inconsistent case-control exposure status, *P*_0_ is the exposure rate of high DII in the control population of about 33%, the expected dietary index-induced exposure to the risk of developing gastric cancer (OR (RR)) is 1.77, *Z*_1−α/2_ is the standardized normal deviation corresponding to the α level, and *Z*_β_ is the standardized normal deviation corresponding to the 1-β level, with the stipulation that α = 0.05, The two-sided test with a degree of certainty 1-β of 0.9, checking the table gives *Z*_1−α/2_ = 1.96 and *Z*_β_ = 1.28, *M* = 265 was calculated, and at least 265 pairs of study participants, totalling 530, were needed for this study.


$$\begin{array}{l} \begin{array}{c} M=\frac{m}{P_{0}\left(1-P_{1}\right)+P_{1}\left(1-P_{0}\right)}\\ m=\frac{{\left[Z_{1-\alpha /2}/2+Z_{\beta }\sqrt{P\left(1-P\right)}\right]}^{2}}{{\left(P-0.5\right)}^{2}}\\ P=OR/\left(1+OR\right)\approx RR/\left(1+RR\right)\\ P_{1}=\left(\begin{array}{c} OR\times P_{0} \end{array}\right)/\left(\begin{array}{c} 1-P_{0}+OR\times P_{0} \end{array}\right) \end{array} \end{array}$$

### 2.2 Questionnair

#### 2.2.1 Food frequency questionnaire (FFQ)

A structured, semi-quantitative FFQ was used to assess the dietary intake of participants. The FFQ covered 78 individual food items or food groups across 13 major categories, including: staple foods (8 items), root vegetables (3 items), pickled/grilled/fried foods (4 items), eggs (2 items), fresh meats (5 items), seafood (5 items), dairy products (4 items), snacks and nuts (4 items), beverages (3 items), soy products (6 items), fresh vegetables (17 items), fresh fruits (12 items), and dried foods (5 items).

Participants were asked to report the average frequency of consumption for each item based on their typical dietary habits over the past 12 months. The FFQ provided nine frequency options, as follows: (1) ≥4 times per day; (2) 2–3 times per day; (3) Once per day; (4) 4–6 times per week; (5) 2–3 times per week; (6) Once per week; (7) 1–3 times per month; (8) Occasionally; (9) Never.

#### 2.2.2 Demographics and lifestyles

In addition to dietary intake, the following covariates were collected:

**General demographic information**, including name, age, sex, height, weight, household income, education level, occupation, and level of daily life stress. Body mass index (BMI) was calculated based on measured height and weight.

**Personal lifestyle habits**, including smoking, alcohol consumption, tea drinking, and coffee intake over the past 12 months. Smoking was defined as smoking ≥1 cigarette per day for more than 6 consecutive months or having smoked ≥150 cigarettes in total. Alcohol drinking was defined as consuming alcohol at least once per week for more than 6 months. Individuals not meeting these criteria were classified as non-smokers or non-drinkers, respectively.

### 2.3 Calculation of the China Dietary Inflammatory Index (CHINA-DII) score

This study referred to the dietary component inflammatory potential scoring method proposed by Shivappa et al. (15). The CHINA-DII calculation process followed the same procedure as the original DII developed by Shivappa et al. The calculation involved five steps, summarized as follows: (1) Dietary intake data of study participants were obtained through dietary questionnaire surveys. (2) For each dietary component, a Z-score representing individual exposure was calculated using the following formula: Z = (individual intake of a dietary component – the mean intake of that component from the Chinese adult dietary intake database)/standard deviation of intake from the Chinese database. (3) To reduce the influence of right-skewed distributions, the calculated Z-scores were then centralized and converted into percentile scores (*q*) ranging from −1 to +1, with 0 as the midpoint. (4) The dietary inflammatory index score for each dietary component was calculated as follows: CHINA-DII (individual component) = *q* × *i*, where “*i*” represents the literature-derived inflammatory effect score of the dietary component and “*q*” represents the centralized percentile value. (5) The total CHINA-DII score was obtained by summing the individual component-specific scores as follows: CHINA-DII = *i*_1_ × *q*_1_ + *i*_2_ × *q*_2_ + …+ *i*_*n*_ × *q*_*n*_.

### 2.4 Statistical analysis

Continuous variables with a normal distribution were described as means and standard deviations (SD), while those not normally distributed were presented as medians and interquartile ranges (IQR: P25, P75). Categorical variables were expressed as frequencies and percentages (*N*, %). Between-group comparisons were performed using the chi-square test for categorical variables and the *t*-test or analysis of variance (ANOVA) for continuous variables, as appropriate.

Participants were categorized into low and high CHINA-DII groups based on the median CHINA-DII score in the control group. Univariate and multivriable logistic regression models were applied to assess the association between CHINA-DII categories and gastric cancer (GC) risk, with odds ratios (ORs) and corresponding 95% confidence intervals (CIs) calculated. Two models were applied to assess the ORs between CHINA-DII and risk of GC: Model 1, unadjusted; Model 2 was adjusted for age group, marital status, smoking, and perceived daily stress level. In addition, CHINA-DII was also analyzed as a continuous variable to evaluate the risk change per one–standard deviation (SD) increase in CHINA-DII score.

To further explore whether the association between CHINA-DII and GC risk varied across subgroups, stratified analyses were conducted based on demographic variables significantly associated with GC risk in univariate analysis. We tested interactions by adding CHINA-DII × subgroup terms to logistic models, with P-interaction determined via likelihood ratio tests. All statistical tests were two-sided, and a *P*-value < 0.05 was considered statistically significant. Data analysis was performed using IBM SPSS Statistics version 26.0.

### 2.5 Ethical considerations

This study was conducted in accordance with the principles of the Declaration of Helsinki and was approved by the Ethics Committee of Fujian Medical University (FJMU No. 2020 [53]). Before participation, the purpose and content of the study were fully explained to the patients, and informed consent was obtained. Participants were free to withdraw from the study at any time if they experienced any discomfort, and refusal to participate had no impact on their medical care. All personal information of the participants was kept strictly confidential at all times.

## 3 Results

### 3.1 Baseline demographics

Table 1 presents the baseline demographics of the study population. A total of 672 participants were included in the analysis, comprising 336 gastric cancer cases and 336 matched controls. The mean age of the participants was 55.31 ± 10.83 years, with cases being significantly older than controls (56.76 ± 10.34 vs. 53.86 ± 11.13 years, *P* < 0.001). Males accounted for 56.5% and females accounted for 44.5%. Most participants were married (90.9%), and over half had an education level of secondary school or below.

**Table 1:** Baseline demography of the study population (*N* = 672).

**Variables**	**Total (*N* = 672)**	**Cases (*N* = 336)**	**Controls (*N* = 336)**	***P* value**
Age, years, mean ± std	55.31 ± 10.83	56.76 ± 10.34	53.86 ± 11.13	<0.001
**Age groups, years**				
≤55	320 (47.6)	132 (39.3)	188 (56.0)	<0.001
>55	352 (52.4)	204 (60.7)	148 (44.0)	
Sex				1.000
Male	380 (56.5)	190 (56.5)	190 (56.5)	
Female	292 (43.5)	146 (43.5)	146 (43.5)	
BMI (kg/m^2^)				0.068
<24	415 (61.8)	196 (58.3)	219 (65.2)	
≥24	257 (38.2)	140 (41.7)	117 (34.8)	
Marital status				0.011
Married	611 (90.9)	315 (93.8)	296 (88.1)	
Single/Seperated/Divorced/Widowed	61 (9.1)	21 (6.2)	40 (11.9)	
Education level				0.157
Primary school or below	276 (41.1)	143 (42.6)	133 (39.6)	
Secondary school	177 (26.3)	90 (26.8)	87 (25.9)	
High school	105 (15.6)	58 (17.3)	47 (14.0)	
College	51 (7.6)	20 (5.9)	31 (9.2)	
University or above	63 (9.4)	25 (7.4)	38 (11.3)	
Occupation				0.361
Farmers/Manual workers	199 (29.6)	99 (29.5)	100 (29.8)	
Other occupations	239 (35.6)	112 (33.3)	127 (37.8)	
Homemakers/Retired/Unemployed	234 (34.8)	125 (37.2)	109 (32.4)	
Average monthly household income, RMB				0.167
<3,000	64 (9.5)	26 (7.7)	38 (11.3)	
3,000–6,000	229 (34.1)	123 (36.6)	106 (31.6)	
>6,000	379 (56.4)	187 (55.7)	192 (57.1)	
Smoking				0.019
Yes	233 (34.7)	131 (39.0)	102 (30.4)	
No	439 (65.3)	205 (61.0)	234 (69.6)	
Alcohol drinking				0.916
Yes	107 (15.9)	53 (15.8)	54 (16.1)	
No	565 (84.1)	283 (84.2)	282 (83.9)	
Daily life stress				<0.001
None/Low	409 (60.9)	227 (67.6)	182 (54.2)	
Moderate/High	263 (39.1)	109 (32.4)	154 (48.8)	
CHINA-DII	−2.11 ± 0.62	−2.04 ± 0.62	−2.18 ± 0.61	0.003

There were no significant differences between the case and control groups in terms of sex, education level, occupation, household income, or alcohol consumption (*P* > 0.05 for all). However, cases were more likely to report moderate or high levels of perceived daily stress (32.4 vs. 48.8%, *P* < 0.001), to be current smokers (39.0 vs. 30.4%, *P* = 0.019), and to be married (93.8 vs. 88.1%, *P* = 0.011). Additionally, the CHINA-DII score was significantly lower in the control group than in the case group (−2.18 ± 0.61 vs. −2.04 ± 0.62, *P* = 0.003).

### 3.2 Comparison of dietary nutrients intake between case and control groups

As shown in Table 2, there were no statistically significant differences between the case and control groups in total energy, protein, carbohydrate, fat, cholesterol, dietary fiber, and most micronutrient intakes (*P* > 0.05). However, the intake of vitamin C and vitamin D was significantly lower in the case group than in the control group. Specifically, the mean intake of vitamin C was 98.77 ± 67.89 mg in cases compared to 119.26 ± 81.58 mg in controls (*P* < 0.001), and the mean intake of vitamin D was 2.10 ± 1.23 μg in cases vs. 2.40 ± 1.14 μg in controls (*P* < 0.001). No significant differences were observed in the intake of β-carotene, vitamin E, or other vitamins and minerals between the two groups.

**Table 2:** Comparison of dietary nutrient intake between the case and control groups.

**Nutrients**	**Total (*N* = 672)**	**Cases (*N* = 336)**	**Controls (*N* = 336)**	***P* value**
Energy (kcal)	1,550.11 ± 513.98	1,539.88 ± 561.70	1,560.33 ± 461.98	0.606
Protein (g)	79.22 ± 32.70	79.21 ± 36.82	79.23 ± 28.03	0.993
Carbohydrates (g)	202.48 ± 69.04	199.84 ± 72.05	205.13 ± 65.89	0.321
Fat (g)	50.54 ± 24.10	50.64 ± 26.32	50.43 ± 21.71	0.912
Saturated fatty acids (g)	12.57 ± 6.07	12.23 ± 6.45	12.91 ± 5.66	0.145
Monounsaturated fatty acids (g)	13.96 ± 7.18	13.68 ± 7.75	14.24 ± 6.57	0.315
Polyunsaturated fatty acids (g)	7.62 ± 4.13	7.68 ± 3.70	7.56 ± 4.52	0.711
Cholesterol (mg)	511.88 ± 267.21	501.33 ± 296.96	522.43 ± 233.93	0.307
Dietary fiber (g)	10.85 ± 6.35	10.61 ± 7.07	11.08 ± 5.53	0.340
Folate (μg)	150.17 ± 84.53	148.11 ± 88.89	152.23 ± 80.00	0.528
Vitamin A (μgRE)	535.47 ± 260.45	525.17 ± 279.62	545.77 ± 239.29	0.305
Vitamin B1 (mg)	0.70 ± 0.29	0.70 ± 0.32	0.70 ± 0.26	0.840
Vitamin B2 (mg)	1.01 ± 0.39	0.99 ± 0.44	1.02 ± 0.34	0.305
Vitamin B3 (mg)	19.69 ± 7.13	19.78 ± 7.83	19.59 ± 6.36	0.740
Vitamin B6 (mg)	0.30 ± 0.23	0.30 ± 0.27	0.31 ± 0.20	0.507
Vitamin C (mg)	109.02 ± 75.70	98.77 ± 67.89	119.26 ± 81.58	**<0.001**
Vitamin D (μg)	2.25 ± 1.20	2.10 ± 1.23	2.40 ± 1.14	**<0.001**
Vitamin E (mg)	10.12 ± 5.59	10.17 ± 6.19	10.07 ± 4.91	0.820
β-carotene (μg)	6,024.43 ± 3,796.34	5,851.12 ± 191.27	4,063.82 ± 221.70	0.237
Fe (mg)	20.31 ± 7.20	20.51 ± 7.90	20.10 ± 6.43	0.461
Zn (mg)	15.10 ± 5.01	15.24 ± 5.35	14.96 ± 4.64	0.449
Mg (mg)	309.31 ± 118.47	309.58 ± 128.63	309.03 ± 107.54	0.952
Se (μg)	69.46 ± 35.82	69.22 ± 39.34	69.70 ± 31.97	0.864

### 3.3 Comparison of dietary nutrients intake between case and control groups

As shown in Table 3, in the univariate analysis, higher intakes of dietary fiber, vitamin B6, vitamin C, and vitamin D were inversely associated with GC risk (*P* < 0.05). However, multivariable logistic regression analysis revealed that only higher intakes of vitamin C and vitamin D were significantly associated with a reduced risk of GC. Individuals in the high-intake group had a 31% lower risk of GC for vitamin C (OR = 0.69, 95% CI: 0.50–0.95, *P* = 0.023) and a 33% lower risk for vitamin D (OR = 0.67, 95% CI: 0.48–0.92, *P* = 0.014), compared to those in the low-intake group.

**Table 3:** Logistic regression analysis for the association between dietary nutrients intake and risk of gastric cancer.

**Nutrient Intake**	**Cases (*N* = 336)**	**Controls (*N* = 336)**	**Univariate logistic regression**		Multivariables logistic regression^*^	
			**OR (95%CI)**	***P* value**	**OR (95%CI)**	***P* value**
**Energy**						
Low intake	176 (52.4)	160 (47.6)	Reference	0.165		
High intake	160 (47.6)	176 (52.4)	0.81 (0.60–1.09)			
**Protein**						
Low intake	179 (53.3)	157 (46.7)	Reference	0.826		
High intake	157 (46.7)	179 (53.3)	0.83 (0.61–1.12)			
**Carbohydrates**						
Low intake	174 (51.8)	162 (48.2)	Reference	0.247		
High intake	162 (48.2)	174 (51.8)	0.84 (0.62–1.13)			
**Fat**						
Low intake	176 (52.4)	160 (47.6)	Reference	0.396		
High intake	160 (47.6)	176 (52.4)	0.88 (0.65–1.19)			
**Saturated fatty acids**						
Low intake	181 (53.9)	155 (46.1)	Reference	0.064		
High intake	155 (46.1)	181 (53.9)	0.75 (0.55–1.02)			
**Monounsaturated fatty acids**						
Low intake	174 (51.8)	162 (48.2)	Reference	0.487		
High intake	162 (48.2)	174 (51.8)	0.90 (0.66–1.22)			
**Polyunsaturated fatty acids**						
Low intake	154 (45.8)	182 (54.2)	Reference	0.053		
High intake	182 (54.2)	154 (45.8)	1.35 (0.99–1.83)			
**Cholesterol**						
Low intake	182 (54.2)	154 (45.8)	Reference	0.075		
High intake	154 (45.8)	182 (54.2)	0.76 (0.56–1.03)			
**Dietary fiber**						
Low intake	185 (55.1)	151 (44.9)	Reference	**0.013**	Reference	0.279
High intake	151 (44.9)	185 (55.1)	0.68 (0.50–0.92)		0.78 (0.50–1.22)	
**Folate**						
Low intake	172 (51.2)	164 (48.8)	Reference	0.396		
High intake	164 (48.8)	172 (51.2)	0.88 (0.65–1.19)			
**Vitamin A**						
Low intake	171 (50.9)	165 (49.1)	Reference	0.758		
High intake	165 (49.1)	171 (50.9)	0.95 (0.71–1.29)			
**Vitamin B1**						
Low intake	178 (53.0)	158 (47.0)	Reference	0.164		
High intake	158 (47.0)	178 (53.0)	0.81 (0.60–1.09)			
**Vitamin B2**						
Low intake	183 (54.5)	153 (45.5)	Reference	0.076		
High intake	153 (45.5)	183 (54.5)	0.76 (0.56–1.03)			
**Vitamin B3**						
Low intake	169 (50.3)	167 (49.7)	Reference	1.000		
High intake	167 (49.7)	169 (50.3)	1.00 (0.74–1.35)			
**Vitamin B6**						
Low intake	181 (53.9)	155 (46.1)	Reference	**0.029**	Reference	0.199
High intake	155 (46.1)	181 (53.9)	0.71 (0.52–0.97)		0.81 (0.58–1.12)	
**Vitamin C**						
Low intake	190 (56.5)	146 (43.5)	Reference	**0.003**	Reference	**0.023**
High intake	146 (43.5)	190 (56.5)	0.62 (0.46–0.85)		0.69 (0.50–0.95)	
**Vitamin D**						
Low intake	195 (58.0)	141 (42.0)	Reference	**0.008**	Reference	**0.014**
High intake	141 (42.0)	195 (58.0)	0.66 (0.49–0.90)		0.67 (0.48–0.92)	
**Vitamin E**						
Low intake	181 (53.9)	155 (46.1)	Reference	0.165		
High intake	155 (46.1)	181 (53.9)	0.81 (0.60–1.09)			
β**-carotene**						
Low intake	170 (50.6)	166 (49.4)	Reference	0.758		
High intake	166 (49.4)	170 (50.6)	0.95 (0.71–1.29)			
**Fe**						
Low intake	166 (49.4)	170 (50.6)	Reference	0.758		
High intake	170 (50.6)	166 (49.4)	1.05 (0.78–1.42)			
**Zn**						
Low intake	168 (50.0)	168 (50.0)	Reference	1.000		
High intake	168 (50.0)	168 (50.0)	1.00 (0.74–1.35)			
**Se**						
Low intake	177 (52.7)	159 (47.3)	Reference	0.105		
High intake	159 (47.3)	177 (52.7)	0.78 (0.57–1.05)			
Mg						0.440
Low intake	172 (51.2)	164 (48.8)	Reference			
High intake	164 (48.8)	172 (51.2)	0.89 (0.66–1.20)			

* Adjusted to fiber, vitamin B6, vitamin C, vitamin D, age group, marital status, smoking, and perceived level of daily life stress.

### 3.4 Association between CHINA-DII and gastric cancer risk

As shown in Table 4, higher CHINA-DII scores were significantly associated with increased GC risk. In the unadjusted model (Model 1), participants in the high CHINA-DII group had a 1.50-fold higher risk of GC compared to those in the low CHINA-DII group (OR = 1.50, 95% CI: 1.11–2.04, *P* = 0.009). This association remained statistically significant after adjusting for age group, marital status, smoking, and perceived daily stress level in Model 2 (OR = 1.45, 95% CI: 1.05–1.99, *P* = 0.023). When CHINA-DII was analyzed as a continuous variable, each one SD increase in score was associated with a 1.26-fold higher risk of GC (OR = 1.26, 95% CI: 1.07–1.48, *P* = 0.006).

**Table 4:** Logistic regression analysis for the association between CHINA-DII score and risk of gastric cancer.

**Model**	**Low CHINA-DII**	**High CHINA-DII**	***P* value**	**Per SD increase**	***P* value**
Model 1^*^	Reference	1.50 (1.11–2.04)	0.009	1.26 (1.25–2.06)	0.003
Model 2^#^	Reference	1.45 (1.05–1.99)	0.023	1.26 (1.07–1.48)	0.006

* Model 1 was unadjusted.

# Model 2 was adjusted for age group, marital status, smoking, and perceived daily stress level.

Subgroup analyses (Table 5) indicated that the positive association between CHINA-DII and GC risk was more pronounced in certain population groups. Specifically, the association was significant among participants aged ≤ 55 years (OR = 2.44, 95% CI: 1.51–3.96, *P* < 0.001), married individuals (OR = 1.41, 95% CI: 1.01–1.96, *P* = 0.044), non-smokers (OR = 1.70, 95% CI: 1.14–2.54, *P* = 0.009), and those with moderate to high levels of perceived daily stress (OR = 2.82, 95% CI: 1.67–4.75, *P* < 0.001). No significant associations were found among participants aged >55 years, smokers, those with low stress levels, or those who were single, separated, divorced, or widowed (*P* > 0.05 for all).

**Table 5:** Stratified analysis of CHINA-DII and gastric cancer risk by demographic characteristics.

**Subgroups**	**Case/control**	**Low CHINA-DII**	High CHINA-DII^*^	***P* value**	***P* for interaction**
Age groups, years					**0.002**
≤55	132/188	Reference	2.44 (1.51–3.96)	**<0.001**	
>55	204/148	Reference	0.92 (0.59–1.44)	0.724	
Marital status					0.570
Married	315/296	Reference	1.41 (1.01–1.96)	**0.044**	
Single/Seperated/Divorced/Widowed	21/40	Reference	1.91 (0.53–6.83)	0.320	
Smoking					0.139
Yes	131/102	Reference	1.09 (0.63–1.88)	0.764	
No	205/234	Reference	1.70 (1.14–2.54)	**0.009**	
Daily life stress					**<0.001**
None/Low	227/182	Reference	0.95 (0.63–1.43)	0.798	
Moderate/High	109/154	Reference	2.82 (1.67–4.75)	**<0.001**	

* Adjusted for age group, marital status, smoking, and perceived daily stress level (excluding stratification factors).

## 4 Discussion

This case-control study aimed to investigate the association between the CHINA-DII, a locally developed measure of dietary inflammatory potential, and the risk of GC. The results demonstrated that higher CHINA-DII scores were significantly associated with an increased risk of GC, and this association remained robust after adjusting for various potential confounders. Additionally, higher intakes of vitamin C and vitamin D were significantly associated with reduced GC risk. Stratified analyses further indicated that the positive association between CHINA-DII and GC risk was more pronounced among younger individuals, non-smokers, married participants, and those with higher levels of perceived daily stress. These findings support the potential role of dietary inflammation in gastric carcinogenesis and highlight the prospects of inflammation-targeted dietary interventions in high-risk populations.

### 4.1 Dietary nutrients and GC

GC is a multifactorial disease influenced by genetic, infectious, environmental, and lifestyle-related factors. Among these, dietary factors have attracted substantial attention in primary prevention due to their modifiability. In this study, 23 dietary nutrients were systematically assessed in relation to GC risk, with findings indicating that higher intakes of vitamin C and vitamin D were significantly associated with reduced risk.

From a biological perspective, vitamin C is a potent antioxidant capable of scavenging free radicals and reducing oxidative stress-induced DNA damage (22). It can also inhibit the endogenous formation of carcinogenic N-nitroso compounds, which play a critical role in gastric carcinogenesis (23). These findings are consistent with previous studies. A meta-analysis of 32 prospective studies reported a 19% reduction in GC risk associated with high vitamin C intake (OR = 0.81, 95% CI: 0.68–0.95), with dose-response analysis suggesting that 65 mg/day might offer optimal protection (24). A case-control study in Korea similarly showed that vitamin C intake was significantly lower among GC patients and inversely associated with GC risk (OR = 0.64, 95% CI: 0.46–0.88) (25).

Vitamin D, particularly its active form 1,25-dihydroxyvitamin D, binds to the vitamin D receptor (VDR) and inhibits tumor cell proliferation while promoting apoptosis (26). A meta-analysis of serum 25(OH)D_3_ levels revealed a significant inverse association with GC incidence, suggesting that sufficient vitamin D status may be protective (27). However, the relationship between dietary vitamin D intake and GC risk remains inconclusive, as some reviews report no statistically significant associations (28).

It is worth noting that in this study, the primary dietary sources of vitamin D were fish, eggs, and red meat. Given that the study population was based in coastal Fujian Province, where fish intake tends to be higher, this regional dietary pattern may have influenced the observed association. Future large-scale, high-quality, multicenter studies across diverse geographic regions are needed to validate the protective role of vitamin D in GC prevention.

### 4.2 CHINA-DII and GC risk

The DII has emerged as a comprehensive indicator of an individual's dietary inflammatory potential and has been implicated in the development of inflammation-related cancers, including GC. Our study found that higher CHINA-DII scores, reflecting more pro-inflammatory diets, were significantly associated with increased GC risk, suggesting that pro-inflammatory dietary patterns may play a critical role in gastric carcinogenesis.

Mechanistically, a high DII score typically reflects a diet rich in pro-inflammatory components such as saturated fats, sugars, and red meats, which can stimulate the production of inflammatory cytokines like TNF-α and IL-6 (29). These cytokines interact with stromal cells, recruit additional inflammatory cells, and maintain a chronic inflammatory microenvironment conducive to tumor proliferation, invasion, angiogenesis, and metastasis. Furthermore, chronic inflammation may reduce the effectiveness of anticancer therapies by altering drug metabolism or vascular permeability and suppressing antitumor immune surveillance, thereby increasing the risk of GC (30).

Several epidemiological studies support a positive association between DII and GC. A prospective cohort study involving over 100,000 participants found a linear relationship between DII and GC risk [OR per tertile decrease in DII in men: 0.73 (0.53–0.99)] (18). Case-control studies from Korea (OR = 1.47, 95%CI: 1.16–1.85) (31), Iran (OR = 3.39, 95%CI: 1.59–7.22) (32), and Brazil (OR = 2.60, 95%CI: 1.16–5.70) (33) (2018–2022) have consistently demonstrated that higher DII scores were associated with significantly higher risk of gastric ulcer or GC. The EPIC study further reported that each one-SD increase in DII was associated with a 25% increase in GC risk, with those in the highest DII quantile having a 1.66-fold higher risk (OR = 1.66, 95%CI: 1.26–2.20) than those in the lowest (17).

### 4.3 Subgroup heterogeneity in the association between CHINA-DII and GC

Stratified analyses in this study revealed potential population heterogeneity in the association between CHINA-DII and GC risk. The association was pronounced in individuals aged ≤ 55 years, consistent with findings from a case-control study in Italy (34). This may reflect a heightened susceptibility of the younger gastric mucosa to dietary inflammatory insults or the greater role of diet in early precancerous processes among those without existing structural abnormalities. Animal studies also suggest that younger organisms exhibit stronger inflammatory responses, possibly due to a less mature mucosal barrier (35). Gastric epithelial cells in younger populations have a faster rate of renewal, and this hyperproliferative state may make proliferating cells more susceptible to oxidative damage when exposed to pro-inflammatory diets, leading to an accumulation of DNA repair errors (36). Moreover, younger individuals may be more likely to adopt high-calorie, high-fat, and processed food diets with stronger pro-inflammatory potential (37, 38). For the younger population, early screening for gastric cancer is recommended, combined with dietary assessment for early identification of high-risk individuals.

Among non-smokers, the association between CHINA-DII and GC risk was also stronger, suggesting that in the absence of a dominant carcinogenic exposure such as smoking, pro-inflammatory diets may exert a more independent effect. In contrast, the strong pro-inflammatory and carcinogenic nature of smoking may mask the marginal effects of diet among smokers (39).

The association was also more prominent among married individuals and those with higher levels of perceived daily stress. Married individuals may have more stable and representative long-term dietary habits (40). Differences in gut microbiota diversity were lower in cohabiting individuals (e.g. mates) than in genetically related separated individuals, suggesting that the shared environment drives microbial convergence and that taxa involved in dietary fiber fermentation are more affected by this effect (41). Psychological stress has been shown to enhance inflammatory responses through activation of the hypothalamic-pituitary-adrenal (HPA) axis and related inflammatory pathways (42), potentially amplifying the negative effects of pro-inflammatory diets. Therefore, it is recommended that those with high levels of perceived stress be screened in conjunction with diet and nutritional interventions implemented accordingly.

These findings suggest the need for more targeted and personalized dietary interventions for GC prevention, particularly among younger adults, those experiencing high psychological stress, and non-smokers with unhealthy dietary patterns. From a public health perspective, CHINA-DII-based dietary strategies can provide dietary guidance to workplace wellness programmes and university health services, facilitate the integration of anti-inflammatory dietary education with mental health services in community clinics, and provide targeted guidance to high-risk populations to increase awareness of anti-inflammatory diets.

### 4.4 Limitations

This study has several limitations. First, as a case-control study, dietary data were retrospectively collected via food frequency questionnaires (FFQs), which may introduce recall bias—especially among patients who might over-report unhealthy dietary behaviors. Second, *Helicobacter pylori* infection, a critical confounder in GC research, was not assessed and may have influenced risk estimates. Its absence in our analysis means we cannot determine whether the observed associations between dietary inflammation and GC are independent of *Helicobacter pylori* infection, or if they might be amplified/attenuated in its presence. While this is a common limitation in nutritional epidemiology studies, future research should ideally combine dietary assessments with *Helicobacter pylori* testing to clarify these relationships. Third, due to incomplete clinical data, GC was not analyzed by histological subtype, limiting the specificity of our findings.

## 5 Conclusion

This case-control study systematically evaluated the associations between dietary nutrient intake, CHINA-DII scores, and gastric cancer risk. The findings indicated that lower intakes of vitamin C and vitamin D, as well as higher CHINA-DII scores—reflecting greater dietary inflammatory potential—were significantly associated with increased GC risk. These results highlight the potential of anti-inflammatory dietary strategies in reducing GC risk.

Our study provides theoretical support for dietary interventions in GC prevention and offers new directions for public health policy. Future efforts should incorporate anti-inflammatory dietary principles into chronic disease prevention frameworks, particularly in high-incidence regions. Community-level nutritional assessments and personalized dietary interventions are recommended to enhance early nutritional risk screening and public awareness regarding the link between diet-induced inflammation and cancer. In addition, it is recommended that dietary surveys be conducted by community health medical personnel when residents undergo annual medical check-ups, while questionnaires for high-risk groups and patients with pre-cancerous lesions should be conducted by hospital specialists, and appropriate measures should be taken according to the results, so as to improve the prevention of gastric cancer in the population. Continued refinement and validation of the CHINA-DII based on local dietary data will be crucial for translating nutritional epidemiology findings into practice and advancing precision prevention efforts in gastric cancer.

## Data Availability

The raw data supporting the conclusions of this article will be made available by the authors, without undue reservation.
